# Beyond the Walls: Intelligent Design for Safer Psychiatric Environments

**DOI:** 10.1192/j.eurpsy.2026.10757

**Published:** 2026-07-31

**Authors:** N. Bouayed Abdelmoula, E. Abdelmoula

**Affiliations:** 1 LR23ES07 Genomics of Signalopathies at the service of Precision Medicine, Medical University of Sfax, Sfax; 2 LR21ES19 UMRAN, National School of Architecture and Urbanism of Tunis; 3 3Doctoral school of architectural sciences and engineering (ED-SIA), Carthage University, Carthage, Tunisia

## Abstract

**Introduction:**

Ensuring security in a psychiatric hospital remains a complex and multifaceted challenge, involving architectural, operational and digital dimensions. In recent years, the integration of Artificial Intelligence (AI) into digital modeling tools such as Building Information Modeling (BIM) and City Information Modeling (CIM) has emerged as a promising avenue to improve safety, responsiveness and inclusivity in mental health institutions.

**Objectives:**

This review of the literature aims to examine the role of AI in improving safety in a psychiatric hospital setting. It focuses on AI-based applications within BIM /CIM systems that contribute to risk prevention, environmental adaptation and the protection of neurodiverse and vulnerable populations.

**Methods:**

A systematic literature review was conducted using the main academic databases. Key words included: Artificial intelligence, psychiatric hospital, BIM, CIM, hospital security.

**Results:**

Our review reveals a growing number of evidence supporting the role of artificial intelligence in improving safety in psychiatric hospital environments. AI technologies, when integrated into BIM and CIM systems, allow the proactive identification of architectural hazards, such as ligation points-through automated spatial analysis. In addition, AI supports trauma-informed design by guiding the incorporation of natural light, biophilic characteristics and acoustic modulation to reduce sensory overload, especially for neurodivergent individuals. AI-powered simulations further optimize patient and staff circulation, improving operational efficiency and responsiveness to emergencies. At the infrastructure level, AI-activated sensor networks facilitate predictive maintenance and continuous monitoring of the environment, thus preventing system failures that could compromise safety. In addition, AI contributes to cybersecurity by detecting anomalies and protecting sensitive patient data in increasingly digitized healthcare environments. Finally, by analyzing behavioral patterns and the various needs of users, AI supports the creation of flexible and inclusive architectural solutions adapted to the cognitive and emotional profiles of psychiatric populations. Together, these applications illustrate how AI is transforming psychiatric institutions into safer, more adaptive and human-centered spaces.

**Conclusions:**

The integration of AI into BIM and CIM frameworks represents a paradigm shift in the design and management of psychiatric healthcare environments. Beyond technical optimization, these tools allow a more proactive, adaptive and ethical approach to safety and inclusivity. However, the literature also highlights the need to continue empirical studies and ethical frameworks to guide the responsible deployment of AI in architectural design of facilities for mental health and psychiatric care.

**Disclosure of Interest:**

None Declared

